# Predicting eyes at risk for rapid glaucoma progression based on an initial visual field test using machine learning

**DOI:** 10.1371/journal.pone.0249856

**Published:** 2021-04-16

**Authors:** Scott R. Shuldiner, Michael V. Boland, Pradeep Y. Ramulu, C. Gustavo De Moraes, Tobias Elze, Jonathan Myers, Louis Pasquale, Sarah Wellik, Jithin Yohannan

**Affiliations:** 1 Wilmer Eye Institute, Johns Hopkins University School of Medicine, Baltimore, MD, United States of America; 2 Massachusetts Eye and Ear, Harvard Medical School, Boston, MA, United States of America; 3 Department of Ophthalmology, Columbia University Medical Center, New York, NY, United States of America; 4 Wills Eye Hospital, Thomas Jefferson University, Philadelphia, PA, United States of America; 5 The Eye and Vision Research Institute of New York Eye and Ear Infirmary at Mount Sinai, Icahn School of Medicine at Mount Sinai School, New York, NY, United States of America; 6 Bascom Palmer Eye Institute, University of Miami, Miami, FL, United States of America; Taipei Medical University, TAIWAN

## Abstract

**Objective:**

To assess whether machine learning algorithms (MLA) can predict eyes that will undergo rapid glaucoma progression based on an initial visual field (VF) test.

**Design:**

Retrospective analysis of longitudinal data.

**Subjects:**

175,786 VFs (22,925 initial VFs) from 14,217 patients who completed ≥5 reliable VFs at academic glaucoma centers were included.

**Methods:**

Summary measures and reliability metrics from the initial VF and age were used to train MLA designed to predict the likelihood of rapid progression. Additionally, the neural network model was trained with point-wise threshold data in addition to summary measures, reliability metrics and age. 80% of eyes were used for a training set and 20% were used as a test set. MLA test set performance was assessed using the area under the receiver operating curve (AUC). Performance of models trained on initial VF data alone was compared to performance of models trained on data from the first two VFs.

**Main outcome measures:**

Accuracy in predicting future rapid progression defined as MD worsening more than 1 dB/year.

**Results:**

1,968 eyes (8.6%) underwent rapid progression. The support vector machine model (AUC 0.72 [95% CI 0.70–0.75]) most accurately predicted rapid progression when trained on initial VF data. Artificial neural network, random forest, logistic regression and naïve Bayes classifiers produced AUC of 0.72, 0.70, 0.69, 0.68 respectively. Models trained on data from the first two VFs performed no better than top models trained on the initial VF alone. Based on the odds ratio (OR) from logistic regression and variable importance plots from the random forest model, older age (OR: 1.41 per 10 year increment [95% CI: 1.34 to 1.08]) and higher pattern standard deviation (OR: 1.31 per 5-dB increment [95% CI: 1.18 to 1.46]) were the variables in the initial VF most strongly associated with rapid progression.

**Conclusions:**

MLA can be used to predict eyes at risk for rapid progression with modest accuracy based on an initial VF test. Incorporating additional clinical data to the current model may offer opportunities to predict patients most likely to rapidly progress with even greater accuracy.

## Introduction

Automated visual field (VF) testing is the clinical standard for diagnosing glaucoma and monitoring progression [1–3] and glaucoma patients vary widely in their rates of progression [4, 5]. It has been estimated that approximately 5–10% of patients with glaucoma will progress rapidly, with rapid progression defined by a VF Mean Deviation (MD) worsening more than1 dB/year [6]. A decrease in the rate of visual field progression by as little as 30% can lead to an improvement in patient quality of life, particularly in patients undergoing rapid progression [7]. Therefore, compared to non-rapid or non-progressors, rapid progressors may have the most to gain from more frequent follow-up and aggressive treatment [8]. Approaches to identify patients who are rapid progressors earlier and with greater accuracy is critical to more individualized treatment to prevent serious visual disability and improve patient outcomes. However, for clinicians relying on individual patient examination, early diagnosis and identification of glaucoma patients at risk for rapid progression is a major challenge.

From a health economics perspective, it has been suggested that while population screening for glaucoma is not cost-effective, targeted screening of high-risk groups may be [9]. Importantly, early identification of patients within these populations who are at high-risk for rapid progression would allow for better allocation of resources, allowing for more cost-effective ways to manage these patients, while avoiding unnecessary testing and intervention in low-risk patients. This is of particular importance in the near term, given the rapid projected growth of glaucoma patients in an aging population that will not be matched by an increase in trained specialists to care for them [10].

Prior work on this topic has focused mostly on more accurate detection of on-going glaucoma progression. Models to detect ongoing progression have been developed using data from the Advanced Glaucoma Intervention Study (AGIS), Early Manifest Glaucoma Trial (EMGT), and Collaborative Initial Glaucoma Treatment Study (CIGTS) [8, 11–15]. However such models detect progression once VF worsening has already occurred and function has already been lost [7, 16].

A smaller number of prior studies have attempted to predict future VF loss based on the trend of prior VF performance. In these studies, linear models of past MD have been shown to achieve greater rates of prediction accuracy for future VF change compared to other measures, such as intraocular pressure, and have been shown to outperform other more complex nonlinear models using past VF data as inputs to predict future VF change [17, 18]. However, these models are frequently plagued by the requirement for a relatively large number of serially obtained VFs as inputs. As patients are frequently lost to follow-up and treatment decisions are often dictated by one or few office visits, early and accurate identification of patients at risk for rapid progression with serial testing may be difficult in real-world clinical settings.

As a field dependent on objective measurement and imaging which generates very large amounts of data, ophthalmology is uniquely primed to benefit from machine learning algorithms (MLAs). Such MLAs have previously been used to diagnose glaucoma and identify progression. As early as 1994, MLAs have been utilized to classify VFs as normal or glaucomatous [19]. Recently, MLAs to distinguish normal VFs from VFs with *preperimetric* glaucoma [20], defined as all VFs from before a diagnosis of manifest glaucoma (presence of glaucoma on VF defined by Anderson-Patella criteria), have achieved area under the receiver operator characteristic (AUC) as high as 0.93 utilizing a deep feed-forward neural network [21]. Additionally, MLAs have been used to classify and detect patterns of VF defects corresponding to retinal nerve fiber layer anatomy [22]. With regard to detecting progression, prior efforts have produced MLAs that have outperformed standardized global indices such as MD and, in some cases, human experts in identifying glaucoma progression [6, 20, 22–31].

However, for MLAs to be useful clinically, they must not only be able to identify progression in serial VF studies, but also predict future progression from baseline or early VF studies. Lee et al. developed a deep learning algorithm capable of predicting future pointwise VFs up to 5.5 years from a single VF measurement, accomplishing a correlation of 0.92 between the MD of predicted and actual future VFs and an average difference of 0.41 dB [32]. However, as most eyes do not undergo VF progression over subsequent measurements [6], the task of accurately predicting future VF data such as MD in the vast majority of patients is less difficult when compared to that of identifying patients most likely to undergo disease progression. Although a step in the right direction, previous efforts have failed to address the task of quickly and accurately identifying patients at risk of rapid VF progression, which is vital for patient care and stratification of healthcare resources. To this end, we sought to compare MLAs to predict risk of rapid glaucoma progression based on the results of a single initial VF test, as well as to elucidate specific factors that may increase risk of rapid progression.

## Methods

Institutional review board approval was obtained at the Johns Hopkins University School of Medicine to use the de-identified dataset and the study adhered to the tenets of the declaration of Helsinki. The institutional review boards of Johns Hopkins University, Columbia University, Harvard University, Mount Sinai School of Medicine, Thomas Jefferson University and University of Miami approved this retrospective study and waived the need for informed consent for the use of these deidentified patient data.

### Data acquisition

A total of 979,203 24–2 VFs were extracted from the Glaucoma Research Network Visual Field Dataset, which included all patients who underwent VF testing with the Humphrey Field Analyzer (HFA; Carl Zeiss Meditec, Dublin, CA) at 6 academic glaucoma centers across the US. Of these, 175,786 VFs from 22,295 eyes met inclusion criteria of having at least five reliable [33] 24–2 VF measurements over the course of follow-up. All VF data, as well as age, was de-identified and extracted.

### Labeling of rapid progressors

For each eye, linear regression was performed to estimate the slope of MD over time using all VF data. Linear regression of MD, which quantifies the overall or generalized sensitivity loss (adjusted for age) averaged across the VF, has been shown to be superior to other global indices in detecting deterioration, particularly among patients undergoing more rapid progression [34]. Rapid progression was defined as worsening of MD by 1 dB/year or more in a given eye [6].

### Statistical analyses

Only data from the initial VF test was used to train MLAs designed to predict the likelihood of future rapid progression. Data were randomly split into a training (80%, 18340 initial VFs/eyes) and held out test (20%, 4585 initial VFs/Eyes). The training and test set were noted to be balanced equally based on percentage of rapid progressors as well as all baseline age and visual field global characteristics (p>0.1 for all comparisons between training and test sets).

Various machine learning algorithms (i.e. support vector machine [35], random forest [36], naïve Bayes [37], and logistic regression [38]) used global visual field indices (MD, PSD, foveal threshold), reliability metrics (false positives, false negatives, fixation losses and test duration) and age as input to predict likelihood of rapid progression. A fully connected neural network [39] was also designed which utilized global indices and age but also included point-wise VF threshold data. Hyper-parameters for each model were optimized to maximize classification accuracy in the validation set. A sensitivity analysis was performed in which a convolutional neural network was developed to take the pointwise data as a spatially oriented grid as described by Lee et. al. [32]. This did not improve model performance over a fully connected neural network. A hybrid model was also created which estimated the likelihood of rapid progression by calculating the mean likelihood of rapid progression of the five models described above. Description of the optimized hyperparameters can be found in **Table 1**.

**Table 1:** Optimized hyperparameters used in various machine learning models.

Machine Learning Algorithm	Optimized Hyperparameters
Support Vector Machine R-Library: e1071	• Kernel: Radial • Class Weights: 8 for Rapid Progressors, 1 for Non-Rapid Progressors
Fully Connected Neural Network R-Library: keras	• Network Architecture: ○ 20% dropout layer→1024 fully connected neuron layer, ReLu activation→: 20% dropout layer→512 fully connected neuron layer, ReLu activation→: 20% dropout layer→ 256 fully connected neuron layer, ReLu activation→128 fully connected neuron layer, ReLu activation→64 fully connected neuron layer, ReLu activation→1 neuro output layer, sigmoid activation • Model Compilation ○ Optimizer: RMS Prop ○ Loss: Binary Cross Entropy • Model Fitting: ○ Validation Split: 20% ○ Number of Epochs: 23 ○ Batch Size: 512 ○ Class Weights: 8 for Rapid Progressors, 1 for Non-Rapid Progressors
Random Forest R-Library: randomForestSRC	• Number of Trees: 2000 • Number of variables randomly selected as candidate for splitting a node: 2

Note: No hyperparameters were needed for logistic regression (R-Library: base R) or Naïve Bayes models (R-Library: e1071).

To assess the effect of including more than the initial VF on MLA performance to predict likelihood of rapid progression, a second set of training was performed. During this training, we included all of the factors mentioned above as inputs, but also included the slope of the first two VFs in an effort to increase model performance.

MLA test set performance was assessed using the area under the receiver operating curve (AUC) for the classification tasks of distinguishing rapid from non-rapid progressors in the testing set only.

In order to understand how the various MLAs were classifying rapid progressors and non-rapid progressors, summary output from MLAs that create summary output including the odds ratio from logistic regression and Breiman-Cutler variable importance plotting from random forest model were assessed.

All statistical analyses were performed in R version 3.5.1 (R Foundation for Statistical Computing, Vienna, Austria).

## Results

A total of 22,925 eyes, from 14,217 patients who underwent VF testing were included in analysis (**Table 2**). Among these, 1,968 (8.6%) underwent rapid progression. Compared to non-rapid progressors, subjects with rapid progression were older (68.6 vs 61.9 years; p<0.01) and rapid progression eyes had worse baseline VF performance measures including lower MD, higher PSD, and lower foveal threshold (p<0.01 for all comparisons). Additionally, rapid progressors tended to have worse visual field reliability metrics than non-rapid progressors (higher percentage of false negative, false positives, more fixation losses, and longer test duration, p<0.01 for all comparisons).

**Table 2:** Demographic of patients with visual field data*.

	Overall	Non-Rapid Progressors	Rapid Progressors
No. of Eyes (%)	22,925	20,957 (91.4)	1,968 (8.6)
No. of Patients	14,217	13,279	1,739
Mean Age of Patients–years, (SD)	62.4 (13.3)	61.9 (13.6)	68.6 (12.8)
Mean Number of VFs per Patient (SD)	7.7 (3.3)	7.7 (3.4)	7.0 (2.6)
Rate of MD Change all VFs dB/Year (SD)	-0.18 (1.2)	-0.03 (1.1)	-1.86 (1.3)
Rate of MD Change First Two VFs dB/Year (SD)*	+2.2 (62.5)	+2.2 (63.4)	+1.9 (51.8)
Mean MD of First VF–decibels, (SD)	-5.2 (6.0)	-5.0 (6.0)	-7.1 (5.7)
Mean PSD of First VF–decibels, (SD)	4.4 (3.6)	4.3 (3.6)	5.6 (3.5)
Mean Foveal Threshold decibels, (SD)	28.2 (9.5)	28.3 (9.5)	27.4 (9.4)
Mean Test Duration minutes (SD)	5.6 (3.2)	5.6 (3.2)	5.8 (3.1)
Mean Percent FN (SD)	12.8 (20.8)	12.3 (20.4)	17.6 (24.4)
Mean Percent FP (SD)	11.8 (19.3)	11.6 (19.3)	13.2 (19.9)
Mean Number of FLs (SD)	0.79 (1.0)	0.78 (1.0)	0.86 (1.0)

*Nonsignificant (p>0.05)

Note: Rapid Progressor is defined as visual Field Mean Deviation Slope <-1dB/yr.

SD = Standard Deviation; MD = Mean Deviation; VF = Visual Field; FN = False Negative; FP = False Positive

**Fig 1:**
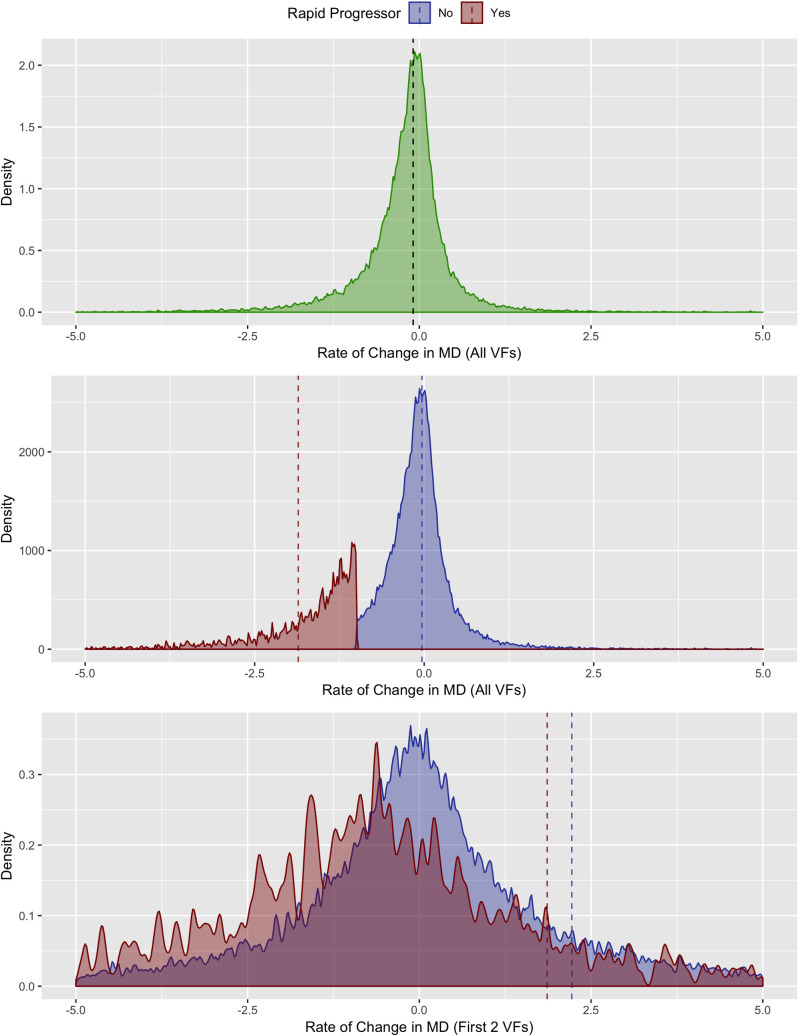
Density plot of rate of MD change. Top panel shows MD slope for all eyes (green). Bottom two panels are subdivided into rates of change for rapid (red) and non-rapid (blue) progressors. Dashed lines indicate mean values.

Distributions for rate of change in MD over time in patients with rapid progression and non-rapid progressors are shown in **Fig 1**. Overall, patients exhibited deterioration over time, resulting in a slightly negative mean MD slope overall (-0.18 [SD 1.2]) with some patients showing improvement/learning effects and others showing variable rates of deterioration. As expected, when using all VFs (5 or more) over time, the distributions of rapid progressors and non-rapid progressors were well separated. By contrast, calculating rate of MD change using the first two VFs poorly differentiated rapid progressors from non-progressors as indicated by substantially greater overlap in the distributions of the two groups.

**Fig 2:**
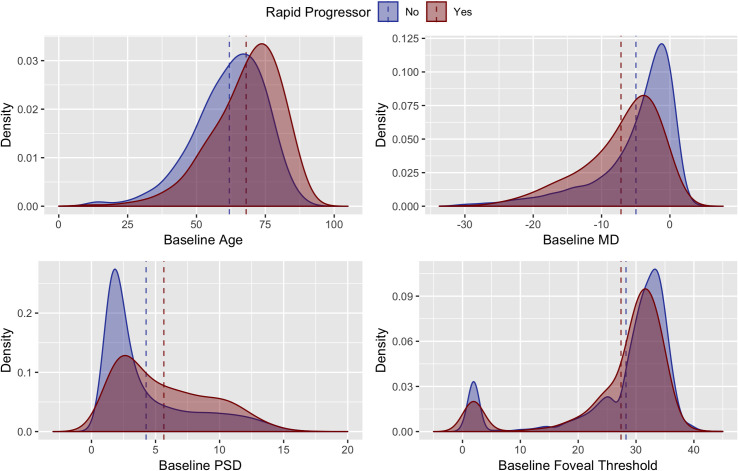
**Density plots of rapid (red) and non-rapid (blue) progressors by age, Mean Deviation (MD), Pattern Standard Deviation (PSD) and foveal threshold at baseline.** Dashed lines indicate mean values for the independent variables in rapid (red) and non-rapid (blue) progressors.

**Fig 3:**
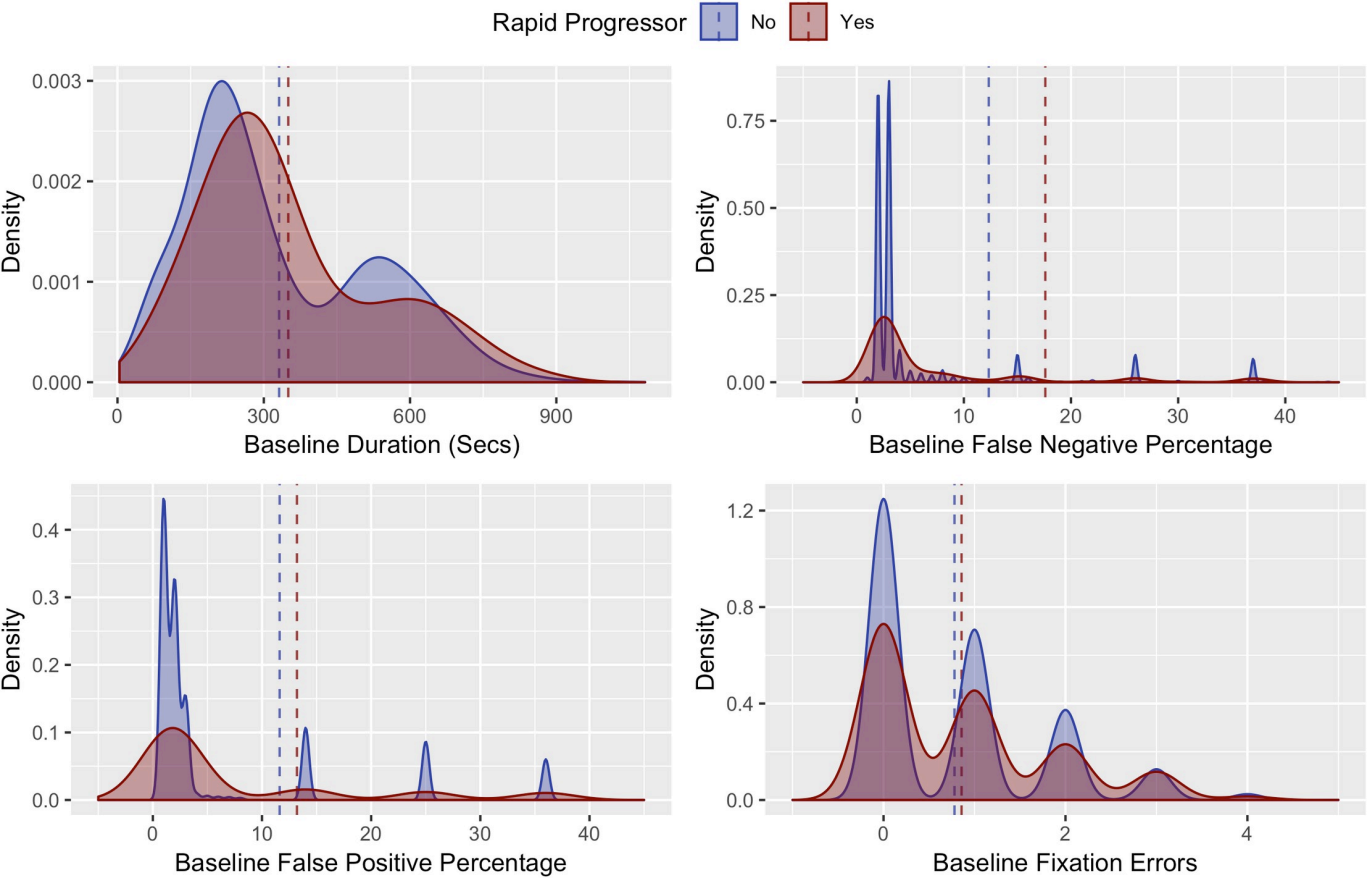
**Density plots of rapid (red) and non-rapid (blue) progressors by VF reliability indices (false negative responses, false positive responses and fixation losses) at baseline.** Dashed lines indicate mean values for the independent variables in rapid (red) and non-rapid (blue) progressors.

**Fig 2** demonstrates the distributions of age and global VF performance measures in rapid progressors and non-rapid progressors. While there were modest differences in baseline age, MD, PSD, and foveal threshold in rapid progressors and non-progressors, there was almost complete overlap of the distributions, suggesting that these global measures were poor individual predictors of rapid progression. When comparing reliability metrics between rapid progressors and non-rapid progressors, rapid progressors had modestly greater false negatives, false positives, fixation errors as well as longer test duration (**Fig 3**).

**Fig 4:**
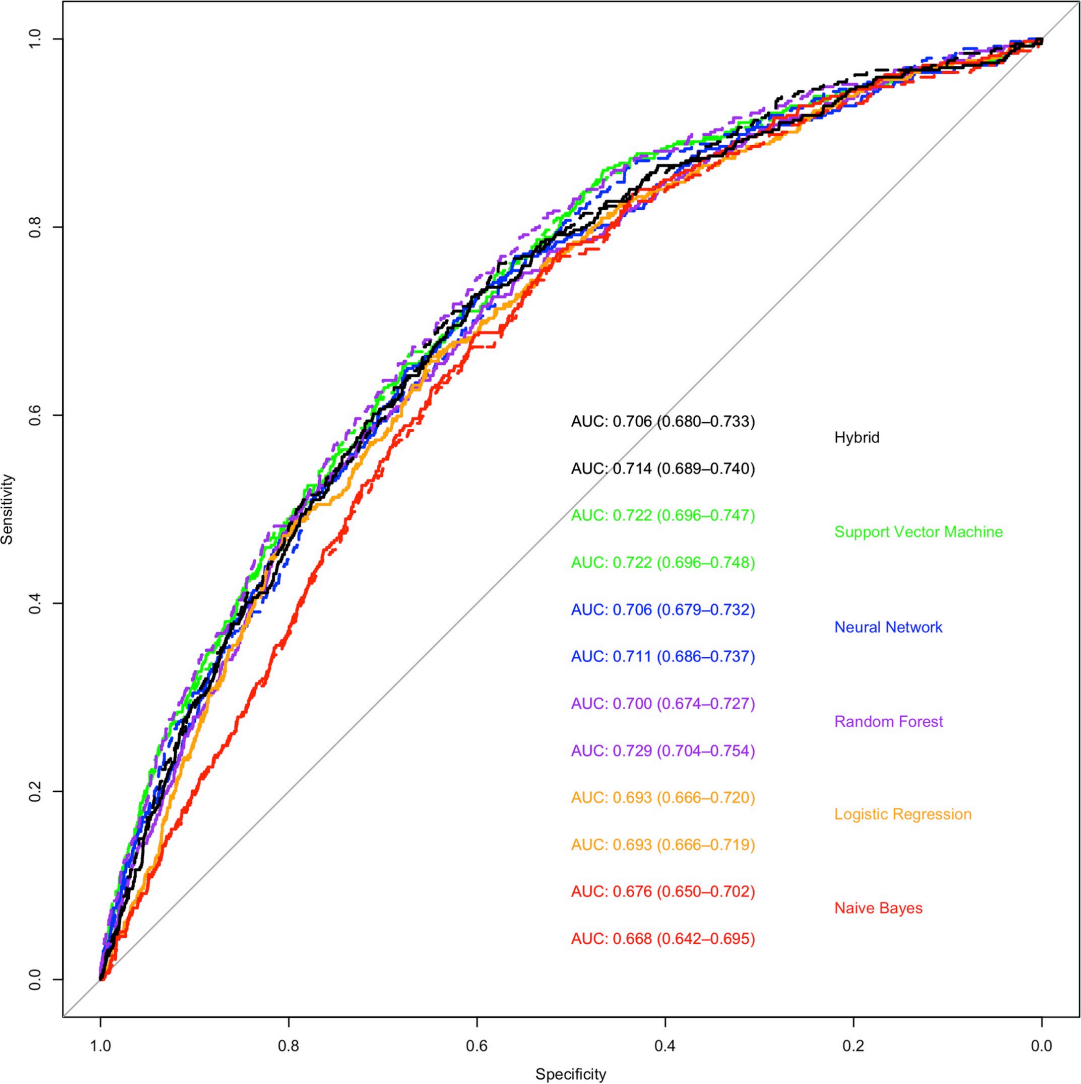
Receiver operating curves that demonstrate the ability of various machine learning methods to classify rapid and non-rapid progressors based on the results of the first or first two visual fields. The colors represent the following machine learning methods; Green- support vector machine; Blue- artificial neural network; Purple- random forest; Orange—logistic regression; Red—naïve Bayes. Black–Hybrid model. The solid lines represent models trained on data from the first VF alone and the dashed line represents models trained on data from the first and second VF. The area under the curve and 95% CI are shown on the bottom right (the top number of each color is model trained on first VF while the bottom number is models trained on first two VFs).

Utilizing the first VF only, the support vector machine model (AUC 0.72) was the most accurate in predicting rapid progression, closely followed by artificial neural network, hybrid, random forest, logistic regression, and naïve Bayes models: AUC 0.72, 0.71, 0.71, 0.70, 0.69, 0.68 respectively (**Fig 4**). When compared statistically, the SVM model was found to have higher AUC than the random-forest, logistic regression and naïve Bayes models (Bonferroni corrected p<0.05 for all comparisons) and equivalent performance to the neural network model (Bonferroni corrected p = 1.00) However, confidence intervals for all AUC curves overlapped, suggesting no real differences in model performance. Of note, utilizing data from the first two VF did not improve accuracy of the models except for the Random forest model (Bonferroni corrected p<0.01 when random-forest trained with one VF compared to Bonferroni corrected random-forest model trained with two VFs). However, when comparing the top performing model (SVM) trained on one VF with the random forest model trained on two VFs, there was no difference in model performance (Bonferroni corrected p = 1). Optimal sensitivity, specificity, positive predictive value and negative predictive value for all models is demonstrated in **Table 3**.

**Table 3:** Model performance metrics.

	Sensitivity	Specificity	Positive Predictive Value	Negative Predictive Value
Support Vector Machine 1 VF	0.65	0.68	0.16	0.95
Support Vector Machine 2 VF	0.66	0.68	0.16	0.96
Neural Network 1 VF	0.49	0.53	0.09	0.92
Neural Network 2 VF	0.54	0.49	0.09	0.92
Random Forest 1 VF	0.72	0.59	0.14	0.96
Random Forest 2 VF	0.67	0.67	0.16	0.96
Logistic Regression 1 VF	0.66	0.65	0.15	0.95
Logistic Regression 2 VF	0.66	0.65	0.15	0.95
Naïve Bayes 1 VF	0.68	0.60	0.14	0.95
Naïve Bayes 2 VF	0.67	0.61	0.14	0.95
Hybrid 2 VF	0.69	0.63	0.15	0.96
Hybrid 2 VF	0.59	0.53	0.15	0.96

VF = Visual Field

A sensitivity analysis was performed using 3 different sets of training/validation and test data and there was no change in MLA performance. Additionally, there was no difference in model performance with 5-fold cross validation. For this method the data are divided into five subsets, four of the subsets are used for training and one is used as a validation set. The error estimation is averaged over five trials to estimate the performance of the model witch may significantly reduce bias [40].

**Fig 5:**
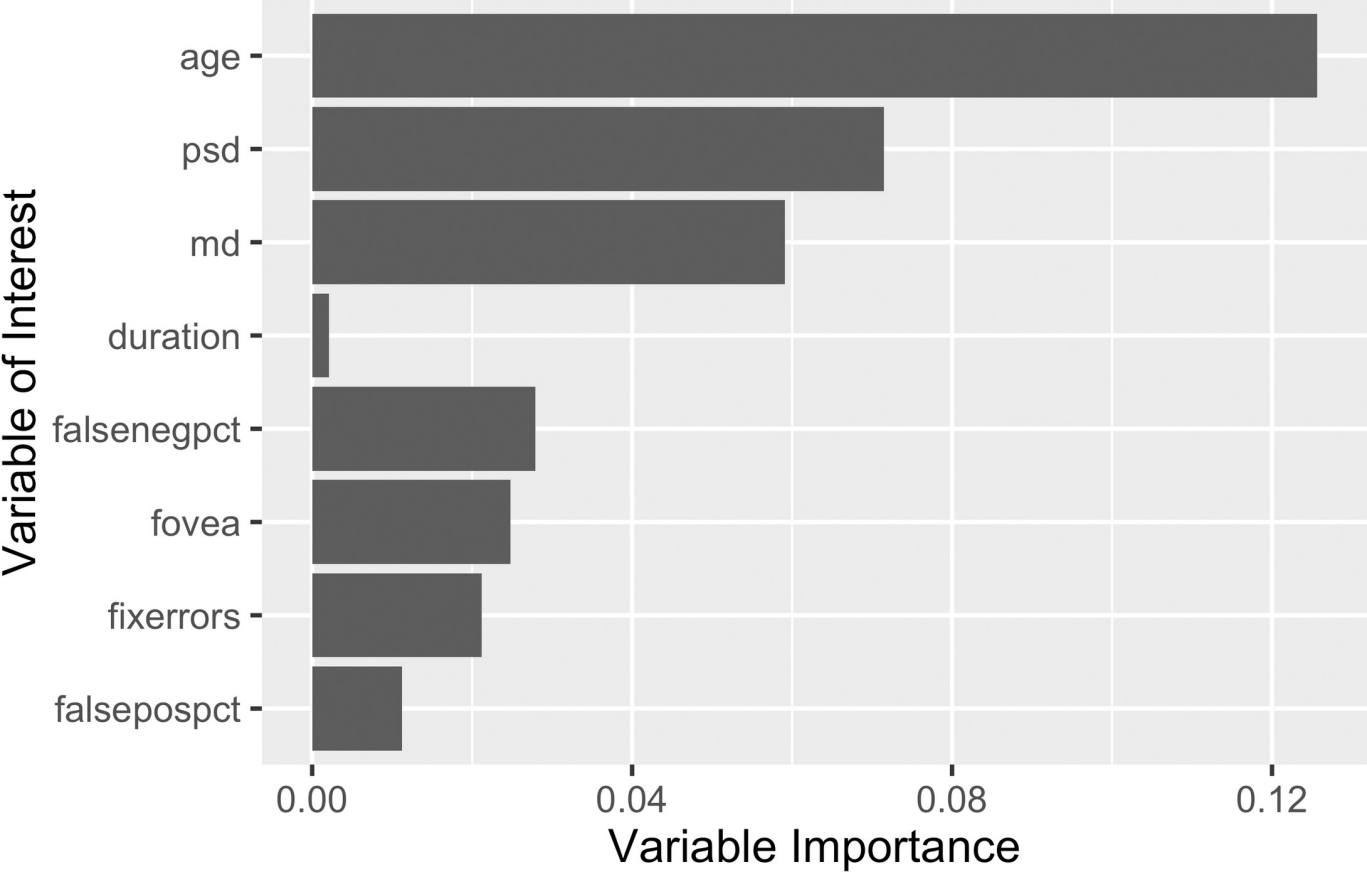
Breiman-Cutler variable importance metrics of the inputs for the random forest model.

In a multivariable logistic regression model, older age, more negative MD, higher PSD and higher false positive and false negative VF testing response rates were significantly associated with a higher odds of rapid progression, each with modest effect sizes (**Table 4**). Plots of variable importance from the random forest regression identified similar predictors of rapid progression (**Fig 5**).

**Table 4:** Factors that influence the odds of being labeled a rapid progressor based on output from the logistic regression model.

Factor at initial VF	Odds Ratio	95% Confidence Interval	P-Value
10-year higher age	1.41	1.34 to 1.48	<0.01
5-dB lower MD	1.08	1.01 to 1.15	0.02
5-dB higher PSD	1.31	1.18 to 1.46	<0.01
5-dB higher foveal threshold*	0.98	0.96 to 1.02	0.38
1-minute greater duration*	1.02	0.99 o 1.02	0.06
10-percent increase in false negative response	1.08	1.06 to 1.10	<0.01
10-percent increase in false positives response	1.04	1.01 to 1.07	0.01
1 additional fixation loss*	1.02	0.96 to 1.02	0.39

*Nonsignificant (p>0.05); dB = decibels

Note: Rapid Progressor is defined as visual Field Mean Deviation Slope <-1dB/yr.

VF = Visual Field; MD = Mean Deviation; PSD = Pattern Standard Deviation

## Discussion

While previous efforts have focused on applying MLAs to identify glaucomatous damage and detect VF progression over time, relatively few studies have endeavored to predict the risk of *future* VF progression. In this study, we utilized MLAs to predict, with a single initial VF test, eyes that subsequently underwent rapid progression. We found that the support vector machine algorithm predicted rapid progression with an AUC of 0.72, while other MLAs performed similarly. Of note, adding data from the first two VFs did not notably improve prediction accuracy over MLAs utilizing only the initial VF. While the accuracy of predictions from our MLAs are modest, they represent a step towards the development clinically relevant tools to identify glaucoma patients at risk for rapid progression earlier, potentially allowing for more effective intervention and better allocation of health resources towards high-risk groups. To our knowledge, no prior work has utilized a longitudinal dataset of this magnitude to demonstrate that a baseline VF can be used to predict risk for rapid progression with relative accuracy. Prior work has utilized data other than VFs, including disc photos and optical coherence tomography (OCT) images and clinical information such as IOP for the early identification of glaucomatous eyes and risk stratification patients for their likelihood of future VF progression [41–44]. However, our MLAs were developed utilizing initial VF data only. Lee et. al. took a pointwise approach in developing deep learning models to predict future VFs [32]. Drawing from 1.7 million individual perimetry points from 32,443 VF tests, their models predicted future VFs in glaucomatous eyes up to 5.5 years in the future, with a correlation of 0.92 between MD of predicted and actual future VFs. While Lee’s model accurately predicts future sensitivity, which is not surprising given that most eyes will not undergo rapid progression, this does not solve the difficult task of identifying the outliers (i.e. those at high risk of rapid progression requiring more aggressive treatment and monitoring).

Our various algorithms performed similarly in identifying patients at risk of rapid progression. For instance, simple logistic regression (AUC 0.69) was not notably less accurate than an artificial neural network (AUC 0.72), suggesting that more complex MLAs may not be necessary to achieve clinically useful accuracy. Additionally, a hybrid model that combined the likelihood estimates from all models did not improve predictive performance. This may be because VFs generate low dimensional data and therefore do not need to be processed by more complex algorithms to achieve optimal classification results.

Of note, we found that adding MD slope from the first two VFs to initial VF data did not improve accuracy. This is not surprising, given that MD differences in these initial tests were highly varied and can possibly be attributed to the learning effect, i.e. patients were still becoming comfortable with VF testing [45]. Also, it should be noted that the mean rate of MD decline in our cohort was -0.18 dB/year, which indicates an overall slow progression of visual changes over time. Conversely, some eyes showed a slightly positive MD slope, which can likely be attributed to learning effects in stable patient (i.e. not true VF improvement).

The factors that were greatest predictors of rapid progression across algorithms were older age, more negative MD, and higher PSD at baseline. These findings are consistent with previous work demonstrating that that risk of progression increases with age, as well as higher PSD and more negative MD at baseline [32, 46–51]. With regard to more negative MD and higher PSD at baseline, it likely these indices represent surrogate markers for more severe glaucoma (i.e., patients who have already worsened), making these patients more likely to continue to undergo progression.

While representing a modest step towards accurate and timely identification of high-risk glaucoma patients, our study has several limitations. Despite their clinical utility, VFs are considered low dimensional data compared to other measures of glaucomatous damage such as optic nerve photos and OCT [52]. VF accuracy is also influenced by diseases other than glaucoma and is plagued by poor reliability due to multiple patient factors, including fatigue, artifacts, and poor reliability indices. In particular, false positives (FP) and false negatives (FN) have been shown to significantly affect VF reliability in patients with established glaucoma, with the degree of impact depending on disease severity [53]. To avoid issues with reliability, we only included reliable VFs in our dataset, though reliability measures (within the allowable range) still predicted rapid progression. Further, while global parameters such as MD can determine an average glaucomatous progression overall, by nature of their averaging out process, they lack localized information regarding disease progression. It should also be noted that this analysis cannot differentiate the causes of rapid progression (i.e., eyes not receiving treatment, eyes non-adherent to treatment or eyes not responding to treatment). However, as our cohort was derived from tertiary ophthalmic care centers, eyes were likely undergoing treatment to prevent glaucoma progression. Finally, although our study utilized a large longitudinal cohort of glaucoma patients with relatively long follow-up from the Glaucoma Research Network visual field dataset, these data may not be generalizable to all glaucoma patients. While there is great promise for implementing MLAs into clinical practice, large longitudinal datasets of glaucoma patients from diverse populations in a variety of clinical settings will be required to achieve clinically useful tools in glaucoma practice.

It should be noted that this study utilized only VF data to identify high-risk patients. The combination of other modalities of data with VF data, such as measurements of nerve fiber layer structure, has been used to detect ongoing glaucoma progression with greater confidence than VF data alone [54, 55]. Further, decisions for management in reality depend on a variety of patient-specific factors in VF data as intraocular pressure, vision in the other eye and life expectancy. Models that do not account for these factors may have somewhat restricted clinical utility. In future work, we hope to combine structural factors such as optic nerve OCT and clinical parameters such as intraocular pressure with visual field data to improve the accuracy of MLAs and achieve AUCs that can be used in a clinical setting (i.e. >0.8) [56].

In summary, we have found MLAs that can, with modest accuracy, identify glaucoma patients at high risk for progression from a single initial VF test. Thus, with further refinement and validation, the MLA approach employed by the present study may serve as an important step in the development of an automated methodology for detection of high-risk glaucoma patients based on an initial encounter, when treatment decisions can be made to alter the course of a patient’s disease. With addition of structural (e.g. optic nerve imaging) and clinical parameters (e.g. IOP) to train MLAs, performance may improve, and MLAs may accurately and rapidly stratify glaucoma patients by progression risk based on a single baseline visit.
